# Characterization of an Immunoglobulin Binding Protein (IbpM) From *Mycoplasma pneumoniae*

**DOI:** 10.3389/fmicb.2020.00685

**Published:** 2020-04-16

**Authors:** Cedric Blötz, Neil Singh, Roger Dumke, Jörg Stülke

**Affiliations:** ^1^Department of General Microbiology, Göttingen Center for Molecular Biosciences, University of Göttingen, Göttingen, Germany; ^2^Medical Faculty Carl Gustav Carus, Institute of Medical Microbiology and Hygiene, Technical University Dresden, Dresden, Germany

**Keywords:** immune evasion, *Mycoplasma pneumoniae*, mollicutes, protein–protein interaction, host–pathogen interaction

## Abstract

Bacteria evolved many ways to invade, colonize and survive in the host tissue. Such complex infection strategies of other bacteria are not present in the cell-wall less *Mycoplasmas*. Due to their strongly reduced genomes, these bacteria have only a minimal metabolism. *Mycoplasma pneumoniae* is a pathogenic bacterium using its virulence repertoire very efficiently, infecting the human lung. *M. pneumoniae* can cause a variety of conditions including fever, inflammation, atypical pneumoniae, and even death. Due to its strongly reduced metabolism, *M. pneumoniae* is dependent on nutrients from the host and aims to persist as long as possible, resulting in chronic diseases. *Mycoplasmas* evolved strategies to subvert the host immune system which involve proteins fending off immunoglobulins (Igs). In this study, we investigated the role of MPN400 as the putative factor responsible for Ig-binding and host immune evasion. MPN400 is a cell-surface localized protein which binds strongly to human IgG, IgA, and IgM. We therefore named the protein MPN400 immunoglobulin binding protein of *Mycoplasma* (IbpM). A strain devoid of IbpM is slightly compromised in cytotoxicity. Taken together, our study indicates that *M. pneumoniae* uses a refined mechanism for immune evasion.

## Introduction

The cell-wall less bacteria of the genus *Mycoplasma* are commensal, opportunistic or pathogenic bacteria that colonize diverse hosts including plants, animals and humans (Parrott et al., 2016). *Mycoplasmas* belong to the group of Mollicutes, which is characterized by their strongly reduced genomes (Dandekar et al., 2000), accompanied by limited metabolic capabilities of these bacteria. The reduction in metabolic pathways is caused by adaptation to their hosts, resulting in the strong dependency on the acquisition of nutrients (Waites and Talkington, 2004; Halbedel et al., 2007). Due to their reduced genomes and their experimental amenability, *Mycoplasma pneumoniae* and *Mycoplasma mycoides* are model organisms for systems and synthetic biology, respectively. Furthermore, both are important pathogens in medical research due to their virulence in their respective hosts, man and cattle. *Mycoplasma* species can cause a broad range of symptoms in various hosts, e.g., fever, inflammation, autoimmune responses, or atypical pneumoniae (Citti and Blanchard, 2013), followed more often by a chronic disease state rather than killing the host (Atkinson et al., 2008). The strong dependency on the host’s survival is a good explanation for the occurrence of chronical diseases caused by *Mycoplasma* infections. Despite their reduced genomes and consequently the limited metabolism *Mycoplasmas* can infect their hosts efficiently.

Remarkably, only few candidate virulence factors of *Mycoplasma* are known or well described. For *M. pneumoniae*, only the community acquired respiratory distress syndrome (CARDS) toxin is described as a typical toxin representative (Kannan and Baseman, 2006; Kannan et al., 2010; Johnson et al., 2011; Becker et al., 2015). Hydrogen peroxide produced during glycerol utilization has been proposed to be the major virulence factor for *M. pneumoniae* and other *Mycoplasmas* (Hames et al., 2009; Blötz and Stülke, 2017). In addition, hydrogen sulfide was also identified to play a significant role in the cytotoxicity of *M. pneumoniae* (Großhennig et al., 2016). Moreover, in some *Mycoplasma* species, e.g., *Mycoplasma fermentans, M. pneumoniae*, *M. genitalium*, or *M. mycoides*, immunomodulatory proteins have been identified (Mühlradt et al., 1997; Into et al., 2004, 2007; Okusawa et al., 2004; Arfi et al., 2016; Campos et al., 2018).

In Gram-positive and Gram-negative bacteria, such as *Yersinia* spp., *Listeria* spp., *Salmonella* spp., or enterohemorrhagic *Escherichia* spp., many different mechanisms which influence the host immune response are well-described (Bhavsar et al., 2007). Overall, the cytadherence or attachment to host cells is a prerequisite for the growth of pathogenic bacteria and concomitant infection (Rottem, 2003; Catrein et al., 2004). The link between attachment and virulence is exemplified by non-adherent *Mycoplasma* mutants which are nearly non-pathogenic (Waldo and Krause, 2006; Chaudhry et al., 2007). *M. pneumoniae* and *M. genitalium* encode a very complex protein network, the attachment organelle, which is responsible for their gliding motility and attachment to human epithelial cells (Balish and Krause, 2006; Kenri et al., 2018; Krause et al., 2018; Seybert et al., 2018). Proteins encoding subunits of this so-called tip structure seem to be responsible for enhanced survival of *M. genitalium* by antigenic and phase variation, a strategy described for many bacteria (van der Woude and Bäumler, 2004; Burgos et al., 2018).

In several Gram-positive bacteria surface proteins are known to bind to human surface proteins. One important class of such binding proteins are immunoglobulin binding proteins (IBPs) (Boyle, 1990; Sidorin and Solov’eva, 2011). The IBPs can bind to different immunoglobulins (Igs) without the requirement of antigen-binding sites. This non-immune binding mechanism is thought to protect bacteria from the action of the complement system. This system is responsible for the phagocyte-independent immune defense in vertebrates, decreasing phagocytosis and finally promoting the evasion of the bacteria from the host’s immune system. IBPs are classified into functional groups according to their ability to bind to human or animal Igs. The most intensively studied IBPs are Protein A (*Staphylococcus aureus*), Protein G (group C *Streptococci*), and the M-protein (group A *Streptococci*) that bind to the Fc region (crystallizable fragment of Igs) (Fischetti, 1989; Sjöbring et al., 1991; Graille et al., 2000), and the Protein L (*Finegoldia magna*) that binds the light chain of Igs (Akerström and Björck, 1989; Graille et al., 2002). Interestingly, the M-protein not only binds to Igs, but it can also bind factor H in serum, thereby blocking the innate immune response (Horstmann et al., 1988).

Recently, IBPs were identified in *M. genitalium* (Protein M) and *Mycoplasma mycoides* subsp. *capri* (*Mycoplasma* immunoglobulin binding protein, MIB) (Grover et al., 2014; Arfi et al., 2016). Interestingly, the 50 kDa IBP from *M. genitalium* differs in its tertiary architecture from all available structures in the Protein Data Bank (PDB). Moreover, its structure is different compared to well-known IBPs (Grover et al., 2014). Protein M and homologs of IBPs from other *Mycoplasmas*, not to be confused with the M-protein from *Streptococci*, seem to have evolved convergently (Frick et al., 1992; Arfi et al., 2016). Regarding the reduced genomes of *Mycoplasmas*, the evolution of a putative immune evasion system is striking. Even more striking, *M. mycoides* possesses multiple paralogous pairs of proteins that bind Igs (MIP) and proteolytically cleave off the variable heavy chain (*Mycoplasma* immunoglobulin protease, MIP). Furthermore, in *Ureaplasma urealyticum* an immunoglobulin A (IgA) specific protease was identified and characterized (Robertson et al., 1984; Spooner et al., 1992). Similarly, an IgG protease was identified in *Mycoplasma synoviae* and *Mycoplasma gallisepticum* (Cizelj et al., 2011). However, the MIB-MIP system present in *M. mycoides* and protein M homologs as found in *M. genitalium*, seem to be conserved mutually exclusive. For *M. pneumoniae*, the presence of a protein M has been predicted (Arfi et al., 2016). The two types of Ig binding proteins do not appear to occur in one genome at the same time. Furthermore, the MIB-MIP system is present in *Mycoplasmas* infecting animals and humans, but not in plant pathogens (Arfi et al., 2016).

In this work, we addressed the localization and function of a putative IBP from *M. pneumoniae*, encoded by *mpn400.* Furthermore, we identified putative proteases and investigated their activity to cleave Igs. Our results demonstrate the surface localization of MPN400 that allows the interaction with external factors. Moreover, we produced recombinant MPN400 in *E. coli* and showed that purified rMPN400 can bind different Igs. A *M. pneumoniae* strain lacking MPN400 exhibits reduced cytotoxicity thus supporting the idea that MPN400 plays an important role for the virulence of *M. pneumoniae*.

## Materials and Methods

### Bacterial Strains, Transformation, and Growth Conditions

The *M. pneumoniae* strains used in this study were *M. pneumoniae* M129 (ATCC 29342) and its isogenic mutant derivative GPM113 (*mpn400*:Tn*4001*). *M. pneumoniae* was grown at 37°C in 175 cm^2^ tissue culture flasks containing 100 ml of modified Hayflick medium with glucose (1%, w/v) as described previously (Halbedel et al., 2004). Surface-attached mycoplasmas were washed four times with phosphate-buffered saline (PBS; pH 7.2). Strains harboring transposon insertions or plasmids were cultivated in the presence of 80 mg/ml gentamicin and/or 2.5 mg/ml tetracycline. Plasmids were introduced into *M. pneumoniae* by electroporation using Gene Pulser (Bio-Rad, Hercules, CA, United States) and the transformants were selected by incubation at 37°C on agar plates containing appropriate antibiotics (Halbedel et al., 2004). For molecular cloning, *Escherichia coli* strain XL1-Blue (Stratagene, San Diego, CA, United States) was grown at 37°C in lysogeny broth (LB) medium containing the appropriate antibiotics (100 mg/ml ampicillin, 5 mg/ml tetracycline).

### Plasmid Construction

Plasmids for the overexpression and purification of MPN400, MPN400 lacking the C-terminus (A446Stop), and MPN641 (negative control) were constructed as follows. The gene of interest was amplified by PCR from M129 *M. pneumoniae* wild type genomic DNA using specifically designed oligonucleotides. The vectors pBSKII(-) (Stratagene) and pGP172 (Merzbacher et al., 2004) were digested with *Kpn*I/*Bam*HI for *mpn400* as well as its truncated version and *Sac*I/*Bam*HI for *mpn641*, respectively. The gene *mpn400* was ligated into pBSKII, resulting in pGP2743. This plasmid served as the template for the multiple mutation reaction (Hames et al., 2005) to replace TGA (opal stop codon in *E. coli*) by TGG (tryptophan) codons. The codon optimized *mpn400* amplicon was used to obtain fragments encoding the protein lacking the *trans*-membrane domain and the C-terminally truncated protein. The products were cloned into pGP172 yielding pGP3215 and pGP3217, respectively. Similarly, the *mpn641* gene was ligated into pGP172. The resulting plasmid was pGP3235. The plasmids were verified by determination of the DNA sequence of the inserts.

### Isolation of Mutant Strains

For the isolation of *M. pneumoniae* mutants, we used an *M. pneumoniae* transposon library carrying insertions of Tn*4001* (Halbedel et al., 2006). The presence of the desired mutant was assayed by a PCR screen using one oligonucleotide that hybridizes to the transposon (directed outward, SH30, 5′ CAATACGCAAACCGCCTC), and a second oligonucleotide specific for the gene of interest (CB37, 5′ GAGAAGAACACTATATCTTTAATAGGTG).

### Southern Blot Analysis

Chromosomal DNA was isolated using the Bacterial DNA Kit (PEQLAB, Erlangen) according to the manufacturer’s instructions. For both strains, M129 and GPM113, cells were grown in T75-flasks and harvested for DNA isolation. 2 μg of each DNA sample was pre-incubated with RNase A (1 μl; 20 mg ml**^–^**^1^) and digested with 3 μl *Sac*I for at least 5 h at 37°C. Digests were separated by agarose gel electrophoresis (1%), transferred onto positively charged nylon membranes (Roche, Munich) and probed with digoxigenin-labeled riboprobes as described earlier (Sambrook et al., 1989; Halbedel et al., 2006).

### Expression and Purification of Recombinant Proteins

The Strep-tagged proteins were overexpressed in and purified from *E. coli* BL21(DE3) as described previously (Schmidl et al., 2011). Briefly, cells were cultivated in two-fold LB medium and the expression (1 l culture, 37°C, 200 rpm, baffled flasks, 3 h) was induced by the addition of 1 mM IPTG to exponentially growing cultures (OD_600_ of 0.6 to 0.8). The cells were pelleted and washed once with buffer W (100 mM Tris-HCl pH8.0, 150 mM NaCl, 1 mM EDTA). Cells lysis was carried out using a French press (18,000 p.s.i., 138,000 kPa, three passes, SLM Aminco, United States), subsequently followed by centrifugation of the crude extracts at 35,000 rpm for 30 min. Purification was done at RT using StrepTrap columns (2 × 5 ml, GE Healthcare). The crude extract was loaded onto the column (flow rate 0.5 ml min**^–^**^1^, max. 0.5 MPa). StrepTraps were washed with buffer W until the 280 nm absorbance reached the base line. We used 2.5 mM D-desthiobiotin for elution (5 ml fractions; flow rate 1–1.5 ml min**^–^**^1^, max. 0.5 MPa). Purification and purity of proteins (>98%) were checked with SDS-PAGE and Colloidal Coomassie staining. Proteins were dialyzed against buffer W using VivaSpin columns (MCO 5 kDa, Sartorius, Göttingen). The pure proteins were frozen in aliquots in liquid nitrogen and stored at −80°C.

### Production of Guinea Pig Polyclonal Antibodies

Polyclonal antisera were produced in guinea pigs (Charles River). The animal experiments were approved by the Ethical Board of Landesdirektion Sachsen, Dresden, Germany (permit no. 24-9168.25-1/2011/1). Primary subcutaneous immunization of animals with recombinant proteins, booster immunizations and serum collection were performed as reported (Thomas et al., 2013).

### Western Blotting

For Western blotting, *M. pneumoniae* GPM113 and the wild type were grown till 95% confluency. Cells were collected and once washed with PBS. Cells were lysed in a tissue lyser with 0.1 mm glass beads (2 × 2.5 min, 30 Hz, cooled block) followed by centrifugation for 10 min at 14,000 rpm and 4°C. From each sample 20 μg total protein was mixed with SDS-loading buffer, boiled for 10 min at 95°C and separated in 12% SDS-PAGE. After electrophoresis, the proteins were transferred to a polyvinylidene difluoride (PVDF) membrane (Bio-Rad) by electroblotting (80 mA/membrane, 1 h). Proteins were detected using antibodies recognizing MPN400 (1:250). The blots were developed with α-guinea pig IgG (Dako; 1:1 000) and visualized by using anti-guinea pig IgG-AP secondary antibodies and the CDP^∗^ detection system (Roche Diagnostics) as described previously (Diethmaier et al., 2011).

### Localization of MPN400

Colony blotting was used to specify the localization of MPN400 as described earlier (Thomas et al., 2013). Briefly, diluted *M. pneumoniae* cells were grown for 10 days on PPLO agar plates and colonies were covered with a nitrocellulose membrane for 5 min. The membrane was dried for 10 min at room temperature, washed and blocked three times (for 10 min, 10% FCS in PBS/Tween). The reactivity of the blotted proteins was tested either with α-MPN400, α-Nox, and α-P14 (1:250 each). The NADH-oxidase (Nox; Pollack et al., 1997; Hagemann et al., 2017) and the C-terminal part of the main P1 adhesin (P14; Schurwanz et al., 2009) were used as cytosolic and surface-localized reference proteins, respectively.

As a further method, mild surface proteolysis of *M. pneumoniae* cells was carried out. *Mycoplasma* cells were grown as described above, harvested, and the protein concentration was adjusted to 200 μg ml**^–^**^1^. The cells were centrifuged for 5 min at 13,000 × *g* and subsequently incubated with 10 μg ml**^–^**^1^, 40 μg ml**^–^**^1^, and 100 μg ml**^–^**^1^ or without trypsin (Sigma) in PBS for 30 min at 37°C. The samples were centrifuged (13,000 × *g*, 10 min) and pellets resuspended in 100 μl protein sample buffer followed by 10 min boiling (95°C) and separation by SDS-PAGE. Subsequently, proteins were blotted onto nitrocellulose membranes by standard procedure. The blots were incubated with α-MPN400, α-Nox, and α-P14 (1:250 each). α-guinea pig IgG (Dako; 1:1,000) was used to detect the proteins.

### Enzyme-Linked Immunosorbent Assay (ELISA)

The binding of immunoglobulins and other human serum proteins by MPN400 was quantified in ELISA experiments. Recombinant MPN641 was used as negative control. MPN641 is a lipoprotein with unknown function and unlikely to bind immunoglobulins. Furthermore, we used human serum albumin and buffer as additional negative controls. MPN400 and a truncated version lacking the predicted C-terminal domain (A446Stop) were used to address Ig binding. Frozen aliquots were thawed and diluted (2, 3, 4, 5, 10, 50 μg ml**^–^**^1^) and subsequently coated onto 96-well plates. The plates were incubated overnight at 4°C and mild shaking (50 rpm). Afterward, wells were washed (buffer + 0.05% Tween20) and blocked (1% skim milk, 1 h, 50 rpm, 4°C) three times, followed by a last washing step. The antibodies [IgA/IgM/IgG (Sigma-Aldrich, Germany); 0.2 ng ml**^–^**^1^] were added to the wells with immobilized recombinant proteins and incubated overnight at 4°C (50 rpm). The wells were washed three times before detection. For detection we used an antibody with affinity to human Igs conjugated to horseradish peroxidase (goat α-Ig HRP detecting IgG, IgM, IgA; Thermo Fisher 0.5 ng ml**^–^**^1^). For detection we incubated wells with 100 μl of α-Ig HRP for 1 h at 4°C (50 rpm). After washing three times, ABTS solution (1-Step ABTS, Thermo Fisher) was added and incubated (20 min, dark, RT). Absorbance detection of bound antibodies was indicated by green color formation, measured in a plate reader (395–415 nm, λ_*max*_ = 405 nm).

### Pulldown of MPN400 Binding Proteins From Human Serum

To isolate MPN400-binding proteins from human serum (Thermo Scientific; H4522), we cultivated *E. coli* strains harboring either pGP3215 to express Strep-MPN400 or the empty vector pGP172. Protein extracts were prepared as described above. StrepTactin columns (CV = 250 μl) were saturated with MPN400 (four columns) or the empty vector crude extract (one column). After extensive washing (10 times 2 ml buffer W), human serum was applied to all columns. For the empty vector column and one with bound MPN400 we used 5 μg of human serum. The remaining MPN400 columns were incubated with 3, 2, and 1 μg of human serum each. After 10 washing steps we eluted the bound proteins four times with buffer W containing D-desthiobiotin (2.5 mM). From elution fractions we used 20 μl for SDS-PAGE analysis, followed by silver staining. Significant bands were further processed by LC-MS/MS analysis.

### Protein Digestion With Trypsin and Protein Identification With LC-MS/MS

Trypsin digestion of proteins was performed as described earlier (Shevchenko et al., 1996). Briefly, the Stage TipStageTip method was used to purify peptides (Rappsilber et al., 2007), which were subsequently separated by reversed-phase liquid chromatography. For analysis an RSLCnano Ultimate 3000 system (Thermo Scientific) followed by mass analysis with an Orbitrap Velos ProHybrid mass spectrometer (Thermo Scientific) was applied as described in more detail elsewhere (Lin et al., 2015; Schmitt et al., 2017). MS/MS2 data processing for peptide analysis and protein identification was performed with the Proteome Discoverer 1.4 software (Thermo Scientific) and the Mascot and SequestHT search algorithms. Proteins identified in empty vector control (pGP172 with human serum) were regarded as unspecific binding and excluded.

### HeLa Cell Cytotoxicity Assay

Infection of HeLa cultures with M. pneumoniae cells was done as described previously (Hames et al., 2009; Schmidl et al., 2010). DMEM medium complemented with 10% FBS was used to grow cells. After 4 days post infection, HeLa cultures were stained with crystal violet (10 min fixation in 10% formalin; 150 μl 0.1% crystal violet solution for 30 min at RT; three times washing) and photographed. For quantification the surviving cells, disruption was carried out with 0.5% SDS solution. The OD_595_ served as indication for cell survival and therefore the cytotoxicity of *M. pneumoniae*.

## Results

### Homology Comparison of Potential Ig-Binding Proteins

**FIGURE 1:**
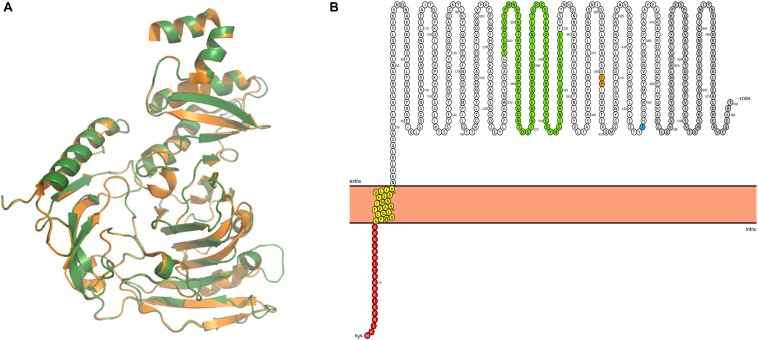
Structural analysis of IbpM (MPN400). **(A)** Structural alignment of the predicted tertiary structure of IbpM from *M. pneumoniae* (green) to the resolved crystal structure of the antibody-binding region of Protein M (PDB: 4NZR, Grover et al., 2014) from *M. genitalium* (orange). The figure was created by PyMOL. **(B)** The ProtterBlot shows the domain architecture of IbpM (using UniProt accession number P75383) anchored in the plasma membrane. Amino acids in red indicate the signal peptide and yellow the predicted transmembrane domain. Colored residues in green indicate a leucine rich region-like domain (LRR domain) and gray the C-terminal disordered domain. Thr insertion point for the mini-transposon is indicated in orange (aa 391–392) and the truncation point for the recombinant mutant in blue (aa 446).

In order to identify possible Ig-binding proteins in *M. pneumoniae*, we searched for homologs of the known mollicute Ig-binding proteins, protein M from *M. genitalium* and MIB from *M. mycoides* in *M. pneumoniae*. For this purpose, we used BLASTp search with standard settings. We identified MPN400, a protein with 55% amino acid identity to protein M (MG281), whereas no protein with similarity to MIB was identified. The alignment of potential Ig-binding proteins in other *Mycoplasma* species revealed only very decent sequence conservation (*Mycoplasma iowae* 19.7%, *Mycoplasma imitans* 19.9%, *Mycoplasma gallisepticum* 21%). For further analysis, structural modeling was used to analyze the structure of MPN400 in comparison to the crystal structure of Protein M of *M. genitalium* (PDB: 4NZR, Grover et al., 2014). The calculated structural alignment was visualized using PYMOL (Omasits et al., 2014). As shown in Figure 1A, the calculated structure seems to be highly similar comparing both IBPs. The sequence and structural comparison revealed a very similar domain architecture as described for Protein M (Figure 1B). The analysis predicts that MPN400 is a membrane protein which is embedded into the membrane via a single *trans*-membrane helix (amino acids 21–40), and that the main part of the protein is exposed to the cell surface.

### Surface Localization of MPN400

**FIGURE 2:**
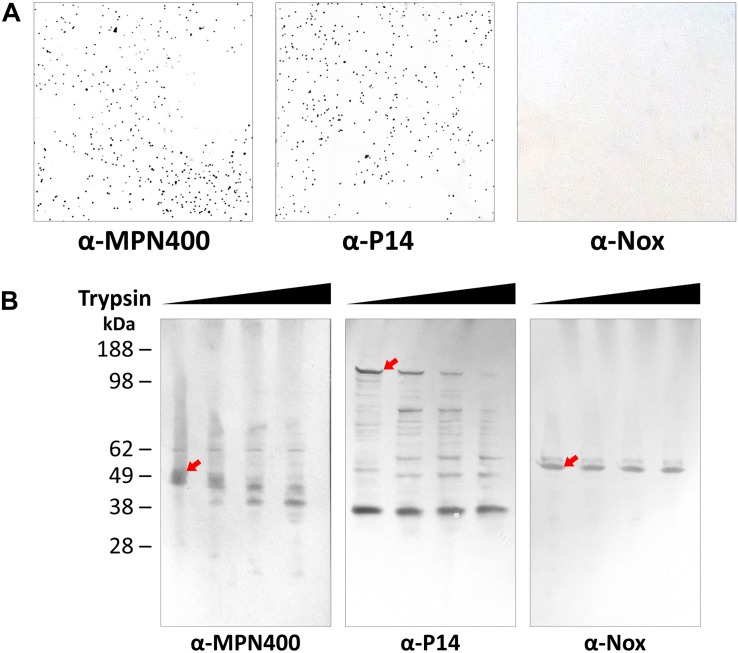
Localization of IbpM (MPN400) on the surface of *M. pneumoniae* cells. **(A)** Results of colony blot of freshly grown *M. pneumoniae* colonies. Blotted colonies were treated with guinea pig α-MPN400, α-P14 (positive control), and α-NADH oxidase (Nox, negative control), respectively. **(B)** Reactivity of SDS-PAGE-separated and blotted whole *M. pneumoniae* proteins after mild treatment with increasing concentrations of trypsin. Lane 1: 0 μg ml^– 1^, lane 2: 10 μg ml^– 1^, lane 3: 40 μg ml^– 1^ and lane 4: 100 μg ml^– 1^ trypsin. Western blots were incubated with guinea pig α-MPN400, α-Nox and α-P14, respectively. The bands corresponding to the non-degraded proteins of interest are highlighted by red arrows. Guinea pig antibodies were detected using rabbit α-guinea pig HRP conjugated antibody.

The analysis described above as well as the described activity of homologs on the cell surface in other *Mycoplasma* species (Grover et al., 2014; Arfi et al., 2016) suggests that MPN400 is also localized to the cell surface. The surface localization was corroborated with colony blotting (Figure 2A) and by mild proteolysis (Figure 2B). In the colony blotting experiment strong signals could be detected for MPN400 and the surface localized protein P14 (positive control), whereas the cytosolic NADH oxidase Nox was not detected exposed to the surface. Further, upon addition of increasing amounts of trypsin, the corresponding bands for surface protein P14 and MPN400 were less prominent and fainter. From this observation, we conclude stronger degradation for these two proteins in contrast to Nox which showed identical bands despite increasing trypsin concentrations. Collectively, these results clearly demonstrate the surface localization of MPN400.

### Isolation of an *mpn400* Mutant

**FIGURE 3:**
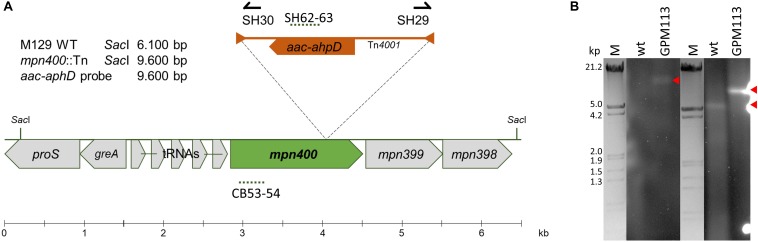
Isolation of a *mpn400* transposon insertion mutant. **(A)** Schematic representation of the genomic region of *mpn400* in *M. pneumoniae* and the transposon insertion site in the *mpn400*:Tn-*4001* mutant GPM113. The location of the probes is indicated by dashed lines. **(B)** Southern blot analysis to confirm single transposon integration using chromosomal DNA of the wild type (wt) and strain GPM113 were digested using *Sac*I. Detection was carried out with a probe specific for the *aac-ahpD* resistance cassette and a probe hybridizing to the gene *mpn400* (right) which is upshifted after transposon integration. The relevant bands are highlighted by red arrows. λ-marker (*Hin*dIII/*Eco*RI) served as a size standard.

A recent analysis of the essential gene set of *M. pneumoniae* revealed that *mpn400* is dispensable (Lluch-Senar et al., 2015). Therefore, we attempted to isolate an mpn400 mutant from an ordered transposon mutant library as described earlier (Halbedel et al., 2006). The pooled *M. pneumoniae* mutants were screened within a PCR reaction to detect the presence of the mini-transposon disrupting *mpn400* (Figure 3A). The successful disruption of *mpn400* and the single transposon integration were verified by Southern blot analysis (Figure 3B). As only one single band could be detected for the *aac-aphD* probe in the mutant (red arrow), we can conclude that the mutant carries a single copy of the transposon which disrupts the *mpn400* gene. For the *mpn400* specific probe a clear shift is visible compared with the wild type signal (indicated by red arrows, Figure 3B); and this shift corresponds to the increased size of the fragment due to transposon integration. The precise integration site was determined by DNA sequencing, showing that *mpn400* is interrupted at its 1178th nucleotide, resulting in a C-terminal truncation of 191 amino acid (of total 582 amino acids). The isolated mutant was named GPM113 (*mpn400*:Tn*4001*).

### Characterization of the *mpn400* Mutant

**FIGURE 4:**
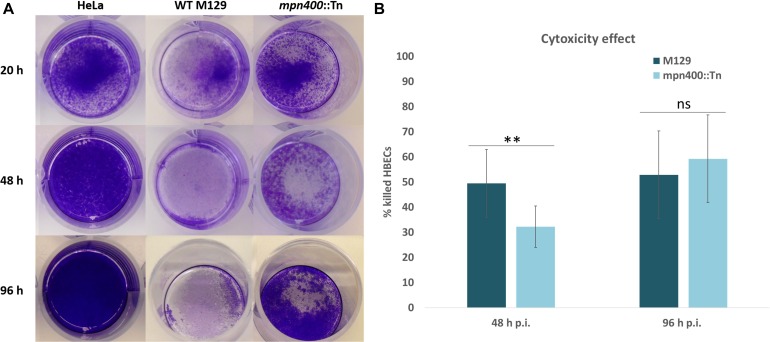
Crystal violet stain of HeLa cells after mycoplasmal infection. Cytotoxicity of *M. pneumoniae* strains toward HeLa cell cultures. Confluent HeLa cell culture without infection or with M129 wild type cells and with GPM113 (*mpn400*:Tn) mutant cells. **(A)** After 20 h, 48 h, and 96 h post infection HeLa cells were stained with crystal violet and photographed. **(B)** OD_595_ measurements were plotted and statistical significance of the cytotoxicities in different strains at the two time points was calculated. For the original data, see Supplementary Data Sheet S1.

When the growth of the mutant strain GPM113 was compared with the wild type M129, no changes could be observed, irrespectively of the carbon source (glucose or glycerol). In agreement with the deletion of the *mpn400* gene, the MPN400 protein was not detectable by colony blotting with GPM113 (data not shown). A Western blot using crude extracts of the wild type and the mutant strain GPM113 showed a faint band with reduced size (∼55 kDa) compared to the wild type MPN400 band at 65 kDa (data not shown). To test the role of MPN400 in virulence, we performed a HeLa cytotoxicity assay (see Figure 4A). This assay revealed a slight, but significant reduction of cytotoxicity of the mutant after 48 h but not 96 h post infection (see Figure 4B). After 48 h post infection wild type cells caused ∼50% cytotoxicity while GPM113 caused less than 35%. Thus, MPN400 plays only a minor role in the direct interaction of M. pneumoniae with the host cells.

### Pulldown of Human Serum Proteins Bound by MPN400

**FIGURE 5:**
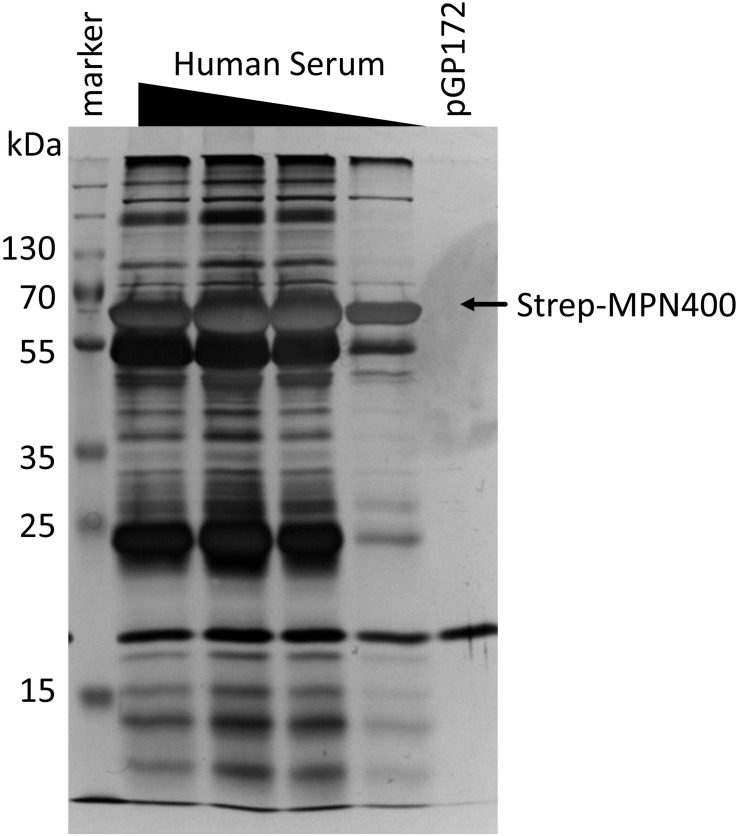
Detection of proteins bound by immobilized MPN400. The binding was performed with recombinant MPN400 (from *E. coli* BL21:pGP3215) immobilized to StrepTactin matrix, which was incubated with different concentrations of human serum (HuSe). Columns were washed extensively, and bound proteins eluted four times with D-desthiobiotin. We used the fractions from elution 3 to analyze the protein content by 12% SDS-PAGE stained with silver. Lane 1, pre-stained protein ladder plus (Thermo Fisher); lanes 2–5, rMPN400 incubated with 5, 3, 2, and 1 μg protein from HuSe, respectively; lane 6, empty vector crude extract from *E. coli* incubated with 5 μg HuSe.

We hypothesized that the main function of MPN400 is an interaction with proteins of the human immune system, in order to allow immune evasion. In order to identify potential interaction partners in human serum in an unbiased manner, we performed a pull-down experiment with MPN400 as the bait. For this purpose, recombinant Strep-tagged MPN400 was immobilized on StrepTactin columns and incubated with different concentrations of human serum. The bound proteins were analyzed with silver stained SDS-PAGE (see Figure 5) and LC-MS/MS analysis. We analyzed proteins larger than 50 kDa for the empty vector control and for MPN400-bound proteins. Among the proteins copurified with MPN400 we identified 1,510 unique peptide-spectrum matches (PSMs) (see Supplementary Table S1 for a complete list). Among the 30 most abundant PSMs were the apolipoprotein B-100 and 17 peptides from immunoglobulins. Non-specific immunoglobulin heavy chains were the most abundant peptides, followed by anti-*Pseudomonas aeruginosa* IgG and anti-rabies virus S057 (Fab) IgG antibodies. Constant regions from the heavy chain were also found along with monoclonal antibodies. Taken together this directed us to test the ability of MPN400 to bind monoclonal and polyclonal antibodies.

### Binding of Immunoglobulins to MPN400

**FIGURE 6:**
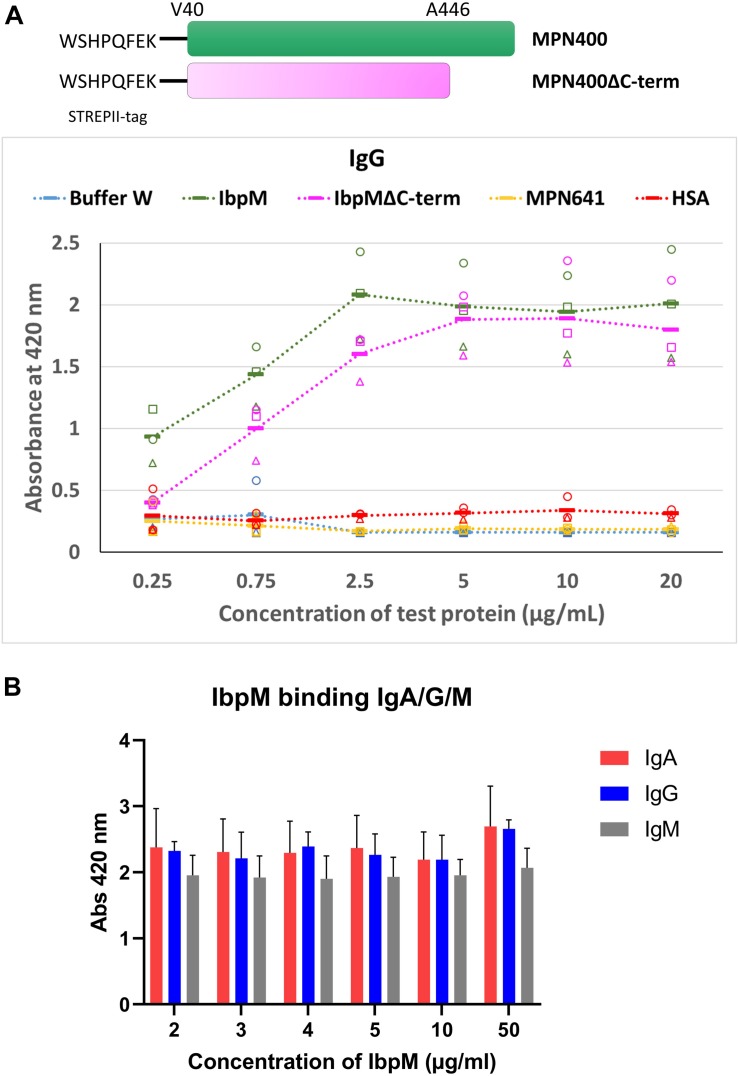
Binding of different Ig’s toward IbpM in an ELISA experiment. **(A)** Purified full-length IbpM, C-terminal truncated IbpM, and human serum albumin (HSA) were coated in different concentrations onto 96-well plates and incubated with IgG, IgA, or IgM. Binding of Ig’s was quantified by the detection with rabbit α-human Ig-AP antibody responsible for ABTS color formation and detection at 420 nm. Squares, circles, and triangles, each indicate a biological replicate mean, each biological replicate itself consisting of three technical replicates. The solid bar represents the average of all measurements. Full-length IbpM in green, truncated IbpM in pink, and HSA in orange. Values from IgG measurement in the graphic are representative as well for IgA and IgM (for graphs of IgA and IgM binding please refer Supplementary Figure S1). **(B)** Binding profiles of full length IbpM to IgA, IgG, and IgM.

To study the binding of Igs to MPN400 in more detail, we performed an enzyme linked immunosorbent assay (ELISA). Recombinantly expressed proteins (MPN400 and negative control lipoprotein MPN641) were coated onto 96-well plates. Coated wells were incubated with different Igs in various concentrations (see Figure 6A). The negative controls (human serum albumin coated or MPN641-coated as well as blank wells with only buffer) showed no binding to the Igs. For MPN400 coated wells we detected strong binding of the tested Igs (IgG, IgA, and IgM) (see Figure 6B for comparative binding across Igs tested, Supplementary Figure S1 for individual binding profiles of the Igs). Thus, MPN400 specifically binds human immunoglobulins.

Studies with the homologous Protein M of *M. genitalium* (Grover et al., 2014) indicated that the C-terminal region of the protein is probably disordered and flexible, which could influence the binding of antibodies. Due to the similarities in the predicted 3D structures for Protein M and MPN400, we also investigated the binding ability of a truncated MPN400 lacking the C-terminal region (residues 486–582) using the ELISA assay. We observed a reduced ability to bind IgG/A/M in the absence of the C-terminal region (see Figure 6). Specifically, at IbpM concentrations up to and including 5 μg/ml, the ability to bind all three antibodies was significantly compromised in recombinant proteins lacking the C-terminal region (Supplementary Figure S1). Thus, contrary to the suggestion that the C-terminal region sterically blocks access to the antigen-binding site, the C-terminal region instead promotes MPN400-antibody interaction or stabilizes it.

**FIGURE 7:**
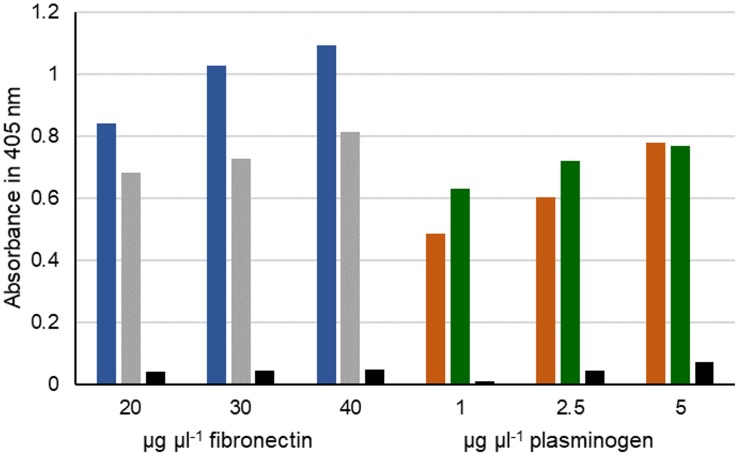
Interaction of fibronectin and plasminogen with IbpM. Purified IbpM (blue and brown) or whole cells of *M. pneumoniae* (gray and green) were coated onto 96-well plates and incubated with different concentrations of fibronectin (left) or plasminogen (right). Interaction of IbpM with the human proteins was quantified with peroxidase-conjugated antibodies detecting the corresponding human proteins. Absorbance was detected at 405 nm. BSA (black bars) served as negative control.

To address the question whether MPN400 binds also to other human proteins, we performed an ELISA assay with fibronectin and plasminogen. Bovine serum albumin (BSA) served as a negative control. As expected, MPN400 did not bind to BSA, whereas we observed binding to fibronectin and plasminogen (see Figure 7). We therefore named *mpn400* the immunoglobulin binding protein gene of *M. pneumoniae* (*ibpM*) and the protein IbpM, respectively.

### Analysis of Putative Immunoglobulin Proteases in *M. pneumoniae*

The protease of the MIB-MIP system (Arfi et al., 2016) was annotated as putative protease bearing a conserved domain of unknown function DUF31. We used the predicted domain DUF31 to scan the *M. pneumoniae* protein sequences for presence of similar domains. The function of this domain still remains elusive, but the amino acid sequence is similar to that of serine proteases. We found sixteen proteins containing a DUF31 domain. Interestingly, 14 of these genes encoding the DUF31 proteins localize in one single cluster of the *M. pneumoniae* genome (*mpn577, mpn580* to *mpn592*, see Table 1). It is worth mentioning, that a similar comparison was done with many other Mollicutes and it seems, that only animal and human pathogenic *Mycoplasmas* encode such DUF31 containing proteins (Arfi et al., 2016). Furthermore, the presence of multiple copies of DUF31 proteins was only found in species lacking the MIB-MIP system (Arfi et al., 2016). This indicates that MIB-MIP and Protein M are mutually exclusive.

**TABLE 1:** *M. pneumoniae* proteins containing DUF31 domains.

Protein	Motif/domain	Size (amino acids)	Location*/information
MPN083	Peptidase S7	533	Membrane-external
MPN084	Peptidase S7	524	Membrane-external
MPN577	Peptidase S7	346	Cytoplasm
MPN580	Peptidase S7	140	Cytoplasm
MPN581		265	Cytoplasm
MPN582	Peptidase S7	439	Membrane-external
MPN583		225	Cytoplasm
MPN584	Peptidase S7	135	Cytoplasm
MPN585		302	Membrane-external
MPN586	GSSGS/peptidase S7	347	Membrane-external
MPN587		150	Membrane-external
MPN588	GSSGS/peptidase S7	531	Membrane-external,^#^
MPN589	Peptidase S7	157	Cytoplasm
MPN590		217	Membrane-external
MPN591	GSSGS/peptidase S7	353	Membrane-external,^ #^
MPN592	GSSGS/peptidase S7	521	Membrane-external,^ #,^^+^

*Predicted; ^ #^Hallamaa et al. (2008); upregulated after A549 contact; ^+^similar to v8 protease (PDB: 1WCZ) from *S. aureus*; GSSGS conserved catalytic serine, all putative proteases encode DUF31 domains.

As MPN592 is similar to the *Staphylococcus aureus* V8 protease, we selected this protein for further analysis. Using the purified protein, we assayed the potential protease activity of this enzyme on IgG, IgA, and IgM as the substrates. However, no activity was detectable, suggesting that MPN592 and probably also the other DUF31 proteases are not involved in immunoglobulin degradation in concert with IbpM.

## Discussion

After successful adherence to epithelial cells, bacteria are exposed to the defense mechanisms of the human host, mainly cell-mediated and humoral immunity. The latter includes antibody-dependent cellular cytotoxicity (ADCC) and activation of complement proteins. The complement can detect antibody–antigen labeled complexes and consequently trigger a degradation cascade for bacterial clearance. To overcome opsonization, phagocytosis, cell lysis and successfully infect host tissue, bacteria must subvert or manipulate the immune system. There are two possibilities, on one hand they could modulate effectors required for detection and degradation of bacterial antigens while on the other hand they could evade the recognition by neutrophils and macrophages in a stealth-like manner (reviewed in Reddick and Alto, 2014; van Avondt et al., 2015).

In this study, we have demonstrated that the surface-localized *M. pneumoniae* IbpM protein binds to human immunoglobulins. Our data show the surface localization of IbpM by two different approaches, colony blot and trypsin digestion. Furthermore, we demonstrate binding of IgG, IgA, and IgM to recombinant IbpM with ELISA experiments. Additionally, we also tested and confirmed the binding capabilities to human plasminogen and fibronectin, which are required for innate immune response against bacterial infection. The C-terminal domain of the IbpM homolog in *M. genitalium* was thought to be not influencing the binding of Igs (Grover et al., 2014) but a truncated version of IbpM showed a reduced binding affinity. However, the precise function of the C-terminal domain remains elusive. As discussed earlier (Grover et al., 2014), after binding of IbpM the C-terminal domain could allow to cover the antigen-binding site and therefore interfere with antigen-antibody interaction. Such a masked Ig could not be detected by the complement or in turn an Ig-wrapped *Mycoplasma* cell could be barely detectable for the host defense mechanism. Lastly, strong binding of IgG by IbpM could invert the effect of opsonization and prevent ADCC (Wang et al., 2015) hindering natural killer cells to eliminate *Mycoplasma* cells. These ideas are in agreement with the chronicity of *Mycoplasma* infection and low mortality rates.

We evaluated proteases in *M. pneumoniae*, based on the described MIB-MIP system from *M. mycoides* (Arfi et al., 2016). Our results revealed the presence of several DUF31 containing proteases in the genome of *M. pneumoniae.* However, specific activity on immunoglobulins was not detectable for the selected protease MPN592, whereas other proteases could not be expressed in *E. coli*. We can thus not exclude the possibility that an Ig-specific protease is encoded in the genome of *M. pneumoniae.* However, it would be interesting to test if any of the DUF31 proteases contributes to the virulence of *M. pneumoniae* even if not involved in Ig degradation. Proteases are known to impact the pathogenicity of bacteria, e.g., the proteases InhA and ClpX of *Bacillus anthracis* contribute to invasion and survival of the pathogen in host tissue (McGillivray et al., 2009; Tonry et al., 2012).

Moreover, we show the binding of fibronectin and plasminogen to IbpM. For surface displayed proteins and lipoproteins in *M. pneumoniae* and other *Mycoplasma* species, the binding of plasminogen and fibronectin was already described (Yavlovich et al., 2004; Henderson et al., 2011; Gründel et al., 2016a, b; Hagemann et al., 2017; Chen et al., 2018; Qi et al., 2018). Neither fibronectin nor plasminogen binding was reported for components of the MIB-MIP system in *M. mycoides*. However, *M. hyopneumoniae* and *M. gallisepticum* MIB both bind plasminogen, but only MIB of *M. hyopneumoniae* also binds fibronectin (Sanderson-Smith et al., 2012). Both organisms possess MIB-MIP homologs. The binding of plasminogen could be mediated by interactions between the Kringle domains of human plasminogen and the leucine-rich repeat (LRR)-like structure in IbpM (see Figure 1B), as the affinity of these domains toward lysine-residues is well known (Bhattacharya et al., 2012; Sanderson-Smith et al., 2012). Furthermore, the binding of IbpM to host cell components could be important for cell invasion (Dallo and Baseman, 2000). Cell migration through tissue barriers involving LRR-containing proteins is already known. For example, *Listeria monocytogenes* uses InlB, a LRR containing protein that induces phagocytosis (Marino et al., 1999; Shen et al., 2000).

The observation of binding of plasminogen to IbpM suggests that *M. pneumoniae* already lost the MIP homolog during evolutionary genome minimization. The protease might be replaced by an Ig-specific protease from the human host, e.g., the plasmin (active form of plasminogen). Plasmin shares 48% amino acid similarity to serine proteases and is known to cleave IgG (Harpel et al., 1989; Chuba, 1994). In addition, the activation of plasminogen to plasmin, was already shown with different surface proteins of *M. pneumoniae* (Gründel et al., 2015; Hagemann et al., 2017). Moreover, binding of plasminogen and fibronectin for themselves could be important. After binding of fibronectin or plasminogen their activated forms can modulate the complement system which would allow *Mycoplasmas* to survive in host tissue (Barthel et al., 2012; Foley et al., 2016). The use of host derived enzymes to circumvent detection and elimination has been described and summarized for Gram-positive and Gram-negative bacteria (Bhattacharya et al., 2012).

## Conclusion

In conclusion, IbpM was confirmed as a surface located multi-immunoglobulin binding protein with affinity for plasminogen and fibronectin. With this, IbpM exhibits all important abilities to subvert the host immune system. Beside other mycoplasmal lipoproteins that facilitate surveillance in the host (Citti and Rosengarten, 1997; Chambaud et al., 1999; Goret et al., 2016), IbpM is a new target for better understanding virulence of *M. pneumoniae.* The functions of IbpM *in vivo* and its implication in virulence, especially potential immunomodulatory effects need to be elucidated in the future. Testing the *mpn400*-deficent mutant in comparison with the wild type in animal models seem to be important.

## Data Availability Statement

All datasets generated for this study are included in the article/Supplementary Material.

## Ethics Statement

The animal study was reviewed and approved by the Ethical Board of Landesdirektion Sachsen, Dresden, Germany (permit no. 24-9168.25-1/2011/1).

## Author Contributions

CB and JS designed the study. CB, NS, and RD performed the experiments and evaluated the data. CB, NS, and JS wrote the manuscript.

## Conflict of Interest

The authors declare that the research was conducted in the absence of any commercial or financial relationships that could be construed as a potential conflict of interest.

## Supplementary Material

FIGURE S1 Ability of IbpM to bind different Ig’s and the effect of the C-terminal region on binding. Using ELISA, purified full-length IbpM was coated in different concentrations onto 96-well plates and incubated withIgG **(A)**, IgA **(B)** or IgM **(C)**. Binding of Igs was quantified by the detection with rabbit α-human Ig -AP antibody responsible for ABTS color formation and detection at 420 nm. The statistical significance of the influence of the C-terminalregion on the binding between IbpM and Igs is indicated by the following scheme: **** is *P* ≤ 0.0001, *** is *P* ≤ 0.001, ** is *P* ≤ 0.01, ns is *P* > 0.05.

TABLE S1 MPN400 pulldown complete protein pulldown list.

DATA SHEET S1 Original Blots Figures 2, 3, 5.
