# Supplementary material for: Characterization of an Immunoglobulin Binding Protein (IbpM) From Mycoplasma pneumoniae
Source: Front Microbiol. 2020 Apr 16;11:685. doi: 10.3389/fmicb.2020.00685 (PMC7176901; doi:10.3389/fmicb.2020.00685)
Supplement: FIGURE S1 — Ability of IbpM to bind different Ig’s and the effect of the C-terminal region on binding. Using ELISA, purified full-length IbpM was coated in different concentrations onto 96-well plates and incubated withIgG (A), IgA (B) or IgM (C). Binding of Igs was quantified by the detection with rabbit α-human Ig -AP antibody responsible for ABTS color formation and detection at 420 nm. The statistical significance of the influence of the C-terminalregion on the binding between IbpM and Igs is indicated by the following scheme: **** is P ≤ 0.0001, *** is P ≤ 0.001, ** is P ≤ 0.01, ns is P > 0.05. [file Image_1.pdf]

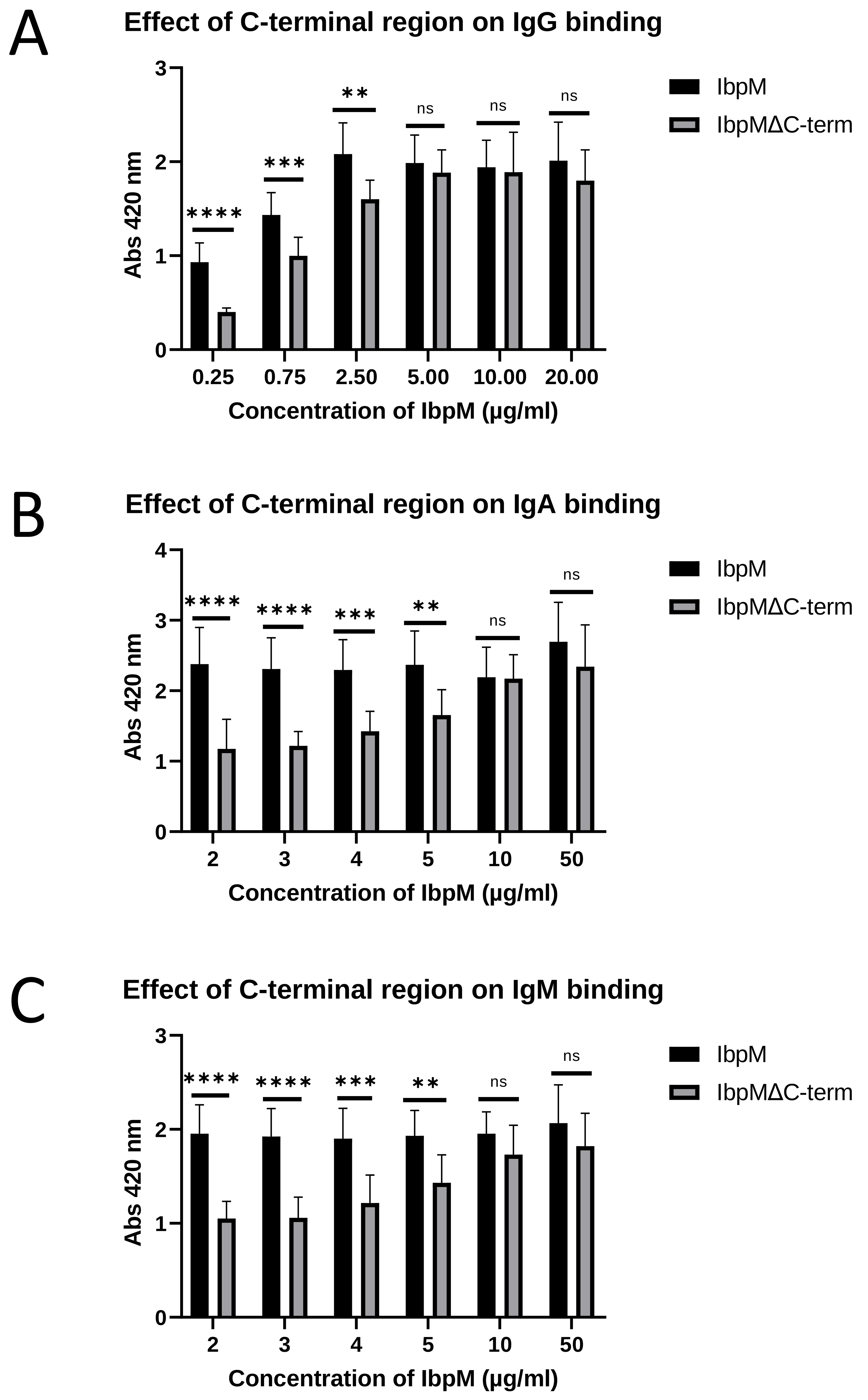

Supplementary Figure S1. Ability of IbpM to bind different Ig's and the effect of the C-terminal region on binding. Using ELISA, purified full-length IbpM was coated in different concentrations onto 96-well plates and incubated with IgG (A), IgA (B) or IgM (C). Binding of Igs was quantified by the detection with rabbit α-human Ig -AP antibody responsible for ABTS color formation and detection at 420 nm. The statistical significance of the influence of the C-terminal region on the binding between IbpM and Igs is indicated by the following scheme: **** is P ≤ 0.0001, *** is P ≤ 0.001, ** is P ≤ 0.01, ns is P > 0.05.
