# Supplementary material for: Characterization of an Immunoglobulin Binding Protein (IbpM) From Mycoplasma pneumoniae
Source: Front Microbiol. 2020 Apr 16;11:685. doi: 10.3389/fmicb.2020.00685 (PMC7176901; doi:10.3389/fmicb.2020.00685)
Supplement: DATA SHEET S1 — Original Blots Figures 2, 3, 5. [file Data_Sheet_1.pdf]

Supplemental information SI 1

Evaluation of HeLa cell viability by Crystal violet staining. After staining, the OD_{595} was determined as an indication for cell density. We performed three biological replicates with three technical replicates each [HeLa 1, HeLa 2, HeLa 3 and similarly for M129 (the strain number for *M.pneumoniae* wild type and GPM-113 which is *M. pneumoniae mpn400* transposon mutant).

1st replicate		
	**OD_{595} after**	
**Culture**	**48 p.i.**	**96 h p.i.**
**Uninfected**		
HeLa 1	7.74	15.48
HeLa 2	12.20	14.70
HeLa 3	9.05	14.80
HeLa control	9.66	14.99
**HeLa infected with**		
M129 - 1	3.52	2.95
M129 - 2	3.49	5.93
M129 - 3	3.34	5.96
M129	3.45	4.95
**HeLa infected with**		
GPM113 - 1	7.41	8.09
GPM113 - 2	7.63	0.69
GPM113 - 3	7.57	4.98
mpn400::Tn	7.54	4.59

2nd replicate		
	**OD_{595} after**	
**Culture**	**48 p.i.**	**96 h p.i.**
**Uninfected**		
HeLa 1	7.04	7.30
HeLa 2	7.20	10.18
HeLa 3	9.50	10.28
HeLa 4	4.62	6.44
HeLa control	7.91	9.25
**HeLa infected with**		
M129 - 1	5.23	7.81
M129 - 2	5.17	4.20
M129 - 3	3.36	3.18
M129 - 4	5.78	6.12
M129	4.89	5.33
**HeLa infected with**		
GPM113 - 1	5.44	7.42
GPM113 - 2	4.40	5.24
GPM113 - 3	5.39	5.27
GPM113 - 4	1.75	3.13
mpn400::Tn	5.08	5.98

3rd replicate		
	**OD_{595} after**	
**Culture**	**48 p.i.**	**96 h p.i.**
**Uninfected**		
HeLa 1	6.57	15.42
HeLa 2	7.76	14.26
HeLa 3	7.87	14.58
HeLa 4	8.31	16.54
HeLa control	7.63	15.20
**HeLa infected with**		
M129 - 1	3.84	9.08
M129 - 2	4.71	6.03
M129 - 3	3.85	8.02
M129	4.13	7.71
**HeLa infected with**		
GPM113 - 1	4.79	4.55
GPM113 - 2	4.73	4.16
GPM113 - 3	4.46	3.60
mpn400::Tn	4.66	4.10
